# Tris(1,10-phenanthroline)cobalt(II) bis­(perrhenate) monohydrate

**DOI:** 10.1107/S1600536810022750

**Published:** 2010-06-18

**Authors:** Samah Toumi Akriche, Zeid Abdellah Al Othman, Mohamed Rzaigui, Refaat Mohamed Mahfouz

**Affiliations:** a Laboratoire de Chimie des Matériaux, Faculté des Sciences de Bizerte, 7021 Zarzouna Bizerte, Tunisia; b Chemistry Department, Faculty of Science, King Saud University, PO Box 2455, Riyadh 11451, Saudi Arabia

## Abstract

In the title compound, [Co(C_12_H_8_N_2_)_3_][ReO_4_]_2_·H_2_O, the Co^II^ atom is coordinated by three 1,10-phenanthroline ligands in a distorted octa­hedral arrangement. In the crystal, the components are linked by O—H⋯O, C—H⋯O and aromatic π–π stacking [shortest centroid–centroid separation = 3.659 (5) Å] inter­actions.

## Related literature

For a related structure and biological background information, see: Li *et al.* (2010 ▶). For geometrical features in related structures, see: Ikotun *et al.* (2008 ▶); Addison *et al.* (1984 ▶).
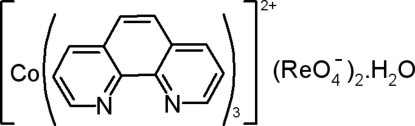


## Experimental

### 

#### Crystal data

[Co(C_12_H_8_N_2_)_3_][ReO_4_]_2_·H_2_O
*M* *_r_* = 1117.96
Triclinic, 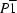

*a* = 10.350 (5) Å
*b* = 13.133 (3) Å
*c* = 14.392 (2) Å
α = 73.58 (2)°
β = 71.18 (2)°
γ = 78.50 (3)°
*V* = 1763.6 (10) Å^3^
*Z* = 2
Ag *K*α radiation
λ = 0.56087 Å
μ = 3.97 mm^−1^
*T* = 293 K
0.17 × 0.15 × 0.13 mm

#### Data collection

Enraf–Nonius CAD-4 diffractometer
24086 measured reflections
17274 independent reflections
8056 reflections with *I* > 2σ(*I*)
*R*_int_ = 0.027
2 standard reflections every 120 min  intensity decay: 3%

#### Refinement

*R*[*F* ^2^ > 2σ(*F* ^2^)] = 0.083
*wR*(*F* ^2^) = 0.244
*S* = 0.98
17274 reflections
493 parameters
3 restraints
H atoms treated by a mixture of independent and constrained refinement
Δρ_max_ = 5.34 e Å^−3^
Δρ_min_ = −5.31 e Å^−3^

### 

Data collection: *CAD-4 EXPRESS* (Enraf–Nonius, 1994 ▶); cell refinement: *CAD-4 EXPRESS*; data reduction: *XCAD4* (Harms & Wocadlo, 1995 ▶); program(s) used to solve structure: *SHELXS86* (Sheldrick, 2008 ▶); program(s) used to refine structure: *SHELXL97* (Sheldrick, 2008 ▶); molecular graphics: *ORTEP-3* (Farrugia, 1997 ▶) and *DIAMOND* (Brandenburg & Putz, 2005 ▶); software used to prepare material for publication: *WinGX* (Farrugia, 1999 ▶).

## Supplementary Material

Crystal structure: contains datablocks I, global. DOI: 10.1107/S1600536810022750/hb5497sup1.cif

Structure factors: contains datablocks I. DOI: 10.1107/S1600536810022750/hb5497Isup2.hkl

Additional supplementary materials:  crystallographic information; 3D view; checkCIF report

## Figures and Tables

**Table 1:** Selected bond lengths (Å)

Re2—O7	1.549 (15)
Re2—O5	1.685 (8)
Re2—O6	1.708 (8)
Re2—O8	1.728 (8)
Re1—O3	1.688 (9)
Re1—O2	1.691 (9)
Re1—O1	1.700 (7)
Re1—O4	1.724 (10)
Co1—N6	2.122 (5)
Co1—N2	2.122 (5)
Co1—N1	2.136 (5)
Co1—N4	2.147 (5)
Co1—N3	2.148 (6)
Co1—N5	2.151 (6)

**Table 2:** Hydrogen-bond geometry (Å, °)

*D*—H⋯*A*	*D*—H	H⋯*A*	*D*⋯*A*	*D*—H⋯*A*
O9—H209⋯O1^i^	0.85 (10)	1.99 (8)	2.802 (13)	157 (19)
O9—H109⋯O8	0.86 (14)	2.26 (17)	2.953 (17)	139 (23)
C15—H15⋯O9^ii^	0.93	2.56	3.271 (14)	134
C33—H33⋯O5^iii^	0.93	2.51	3.116 (12)	123
C5—H5⋯O4^iv^	0.93	2.45	3.309 (13)	154
C33—H33⋯O5^iii^	0.93	2.51	3.116 (12)	123

Symmetry codes: (i) 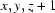; (ii) 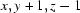; (iii) 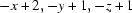; (iv) 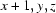.

**Fig. 1.**
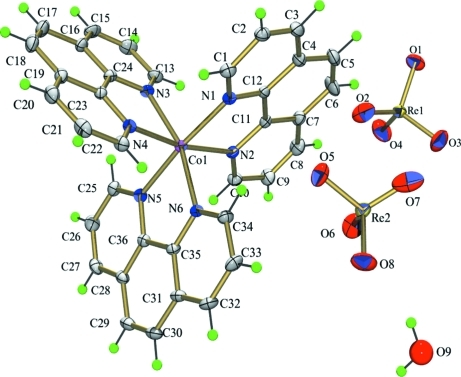
The structure of (I) with displacement ellipsoids drawn at the 30% probability level.

**Fig. 2.**
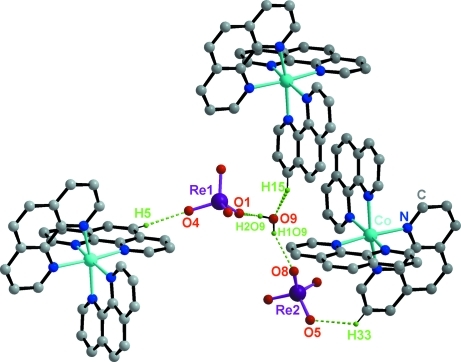
Hydrogen-bonding interactions in (I); the H-atoms not involved in H-bonding are omitted.

## supplementary crystallographic information

### Comment

As part of our exploration of metal complexes with possible biological applications (Li *et al.*,  2010), we now report the structure of the title compound, (I)> It contains one cationic mononuclear species [Co(phen)_3_]^2+^, two perrhenate anions, and one lattice water molecule (Fig. 1). The unique crystallographically independent Co^II^ exhibits a distorted octahedral environment with a τ value of 0.05 (2) calaculated using the approach of Reedijk and co-workers (Addison *et al.*, 1984). Six nitrogen donors from three bidendate phen ligands are coordinated to Co^II^. The Co^II^ is slightly displaced from the octahedron centroid by 0.053 (1) Å. The structural data are in good agreement with those of the cobalt(II) complexes which exhibit a similar geometry (Li *et al.*, 2010). The phen molecules are nearly planar (mean deviation is 0.048 (3) Å). The mean dihedral angle between the two pyridyl planes being 3.0 (2)°. The intra-ring C—N and C—C bond distances have respectively the usual mean values 1,344 (8) Å) and 1.397 (11)Å (Ikotun *et al.*, 2008). The angles subtended by the bidentate phen ligand at the cobalt atom are comparable with a mean value 77,9(2)°. The charges are counterbalanced by uncoordinated perrhenate anions which are connected through hydrogen bonds (C—H ···O) to the coordinated phen molecules (Table 1 and Fig. 2). The mononuclear units are also interconnected through intermolecular hydrogen bonds involving the uncoordinated water molecule and perrhenate oxygen atoms (Table 1) to form trinuclear unit which is further stabilized by the inter-phen ring *p*-p stacking (Fig. 2). The mean interplanar distance is 3.719 Å and the angle made by ring normal and the vector between the ring centroids is 7.95° (mean value).

### Experimental

An aqueous solution (15 ml) of NH_4_ReO_4_ (0.54 g; 2 mmol) was slowly added under stirring to a mixture of ethanol (5 ml) and water (15 ml) containing phen (0,42 g; 3 mmol) and CoCl_2_ (0.2 g; 1 mmol). The purple solution was left in air for a week and pink prisms of (I) were recovered.

### Refinement

The water H atoms were located in a difference map and freely refined. All H atoms attached to C atoms were fixed geometrically and treated as riding, with C—H = 0.93 Å and with *U*_iso_(H) = 1.2Ueq(C).

### Crystal data

**Table:**

[Co(C_12_H_8_N_2_)_3_][ReO_4_]_2_·H_2_O	*Z* = 2
*M**_r_* = 1117.96	*F*(000) = 1066
Triclinic, *P*1	*D*_x_ = 2.105 Mg m^−^^3^
Hall symbol: -P 1	Ag *K*α radiation, λ = 0.56087 Å
*a* = 10.350 (5) Å	Cell parameters from 25 reflections
*b* = 13.133 (3) Å	θ = 9–11°
*c* = 14.392 (2) Å	µ = 3.97 mm^−^^1^
α = 73.58 (2)°	*T* = 293 K
β = 71.18 (2)°	Prism, pink
γ = 78.50 (3)°	0.17 × 0.15 × 0.13 mm
*V* = 1763.6 (10) Å^3^	

### Data collection

**Table:**

Enraf–Nonius CAD-4 diffractometer	*R*_int_ = 0.027
Radiation source: fine-focus sealed tube	θ_max_ = 28.0°, θ_min_ = 2.2°
graphite	*h* = −17→17
non–profiled ω scans	*k* = −21→21
24086 measured reflections	*l* = −6→24
17274 independent reflections	2 standard reflections every 120 min
8056 reflections with *I* > 2σ(*I*)	intensity decay: 3%

### Refinement

**Table:**

Refinement on *F*^2^	Primary atom site location: structure-invariant direct methods
Least-squares matrix: full	Secondary atom site location: difference Fourier map
*R*[*F*^2^ > 2σ(*F*^2^)] = 0.083	Hydrogen site location: inferred from neighbouring sites
*wR*(*F*^2^) = 0.244	H atoms treated by a mixture of independent and constrained refinement
*S* = 0.98	*w* = 1/[σ^2^(*F*_o_^2^) + (0.1366*P*)^2^] where *P* = (*F*_o_^2^ + 2*F*_c_^2^)/3
17274 reflections	(Δ/σ)_max_ = 0.035
493 parameters	Δρ_max_ = 5.34 e Å^−^^3^
3 restraints	Δρ_min_ = −5.31 e Å^−^^3^

### Special details

**Table:**

Geometry. All e.s.d.'s (except the e.s.d. in the dihedral angle between two l.s. planes) are estimated using the full covariance matrix. The cell e.s.d.'s are taken into account individually in the estimation of e.s.d.'s in distances, angles and torsion angles; correlations between e.s.d.'s in cell parameters are only used when they are defined by crystal symmetry. An approximate (isotropic) treatment of cell e.s.d.'s is used for estimating e.s.d.'s involving l.s. planes.
Refinement. Refinement of *F*^2^ against ALL reflections. The weighted *R*-factor *wR* and goodness of fit *S* are based on *F*^2^, conventional *R*-factors *R* are based on *F*, with *F* set to zero for negative *F*^2^. The threshold expression of *F*^2^ > σ(*F*^2^) is used only for calculating *R*-factors(gt) *etc*. and is not relevant to the choice of reflections for refinement. *R*-factors based on *F*^2^ are statistically about twice as large as those based on *F*, and *R*- factors based on ALL data will be even larger.

### Fractional atomic coordinates and isotropic or equivalent isotropic displacement parameters (Å^2^)

**Table:**

	*x*	*y*	*z*	*U*_iso_*/*U*_eq_	
Re2	0.81148 (4)	0.32794 (3)	0.47046 (3)	0.05237 (11)	
Re1	0.42492 (4)	0.26252 (3)	0.05290 (2)	0.05389 (11)	
Co1	0.77573 (8)	0.78259 (7)	0.30120 (6)	0.03267 (17)	
N1	0.9503 (5)	0.6807 (4)	0.2371 (4)	0.0360 (11)	
N2	0.6819 (5)	0.6648 (4)	0.2795 (4)	0.0373 (11)	
N3	0.7832 (6)	0.8921 (5)	0.1575 (4)	0.0386 (11)	
N4	0.8990 (5)	0.8977 (4)	0.2982 (4)	0.0357 (10)	
N5	0.5837 (5)	0.8693 (5)	0.3666 (5)	0.0383 (11)	
N6	0.7463 (5)	0.7149 (4)	0.4579 (4)	0.0353 (10)	
O1	0.5215 (9)	0.2398 (7)	−0.0613 (6)	0.087 (2)	
O2	0.4816 (12)	0.3588 (9)	0.0815 (7)	0.119 (4)	
O3	0.4311 (14)	0.1461 (8)	0.1403 (7)	0.125 (4)	
O4	0.2576 (10)	0.2997 (10)	0.0475 (10)	0.123 (4)	
O5	0.8842 (9)	0.4410 (6)	0.4074 (8)	0.089 (3)	
O6	0.6492 (9)	0.3431 (8)	0.4588 (8)	0.102 (3)	
O7	0.9060 (11)	0.2337 (12)	0.4291 (8)	0.132 (4)	
O8	0.7999 (11)	0.3087 (9)	0.5966 (6)	0.113 (4)	
O9	0.6895 (13)	0.1463 (10)	0.7811 (8)	0.118 (3)	
H109	0.69 (2)	0.180 (17)	0.721 (4)	0.230*	
H209	0.622 (12)	0.166 (14)	0.828 (8)	0.153*	
C1	1.0837 (7)	0.6876 (6)	0.2185 (6)	0.0453 (15)	
H1	1.1092	0.7374	0.2421	0.054*	
C2	1.1877 (7)	0.6209 (6)	0.1635 (6)	0.0485 (17)	
H2	1.2798	0.6291	0.1495	0.058*	
C3	1.1533 (8)	0.5461 (7)	0.1318 (7)	0.0541 (19)	
H3	1.2216	0.5025	0.0959	0.065*	
C4	1.0142 (8)	0.5333 (6)	0.1528 (6)	0.0463 (15)	
C5	0.9697 (9)	0.4542 (7)	0.1245 (8)	0.062 (2)	
H5	1.0338	0.4069	0.0904	0.074*	
C6	0.8313 (10)	0.4475 (8)	0.1478 (9)	0.070 (3)	
H6	0.8038	0.3948	0.1291	0.084*	
C7	0.7289 (9)	0.5179 (6)	0.1990 (6)	0.0483 (16)	
C8	0.5883 (10)	0.5129 (7)	0.2260 (8)	0.061 (2)	
H8	0.5557	0.4645	0.2053	0.073*	
C9	0.5006 (9)	0.5762 (7)	0.2806 (8)	0.060 (2)	
H9	0.4071	0.5694	0.3022	0.073*	
C10	0.5501 (7)	0.6541 (6)	0.3057 (6)	0.0476 (16)	
H10	0.4873	0.6999	0.3423	0.057*	
C11	0.7723 (7)	0.5954 (5)	0.2277 (5)	0.0362 (12)	
C12	0.9166 (6)	0.6051 (5)	0.2043 (5)	0.0345 (12)	
C13	0.7240 (8)	0.8886 (7)	0.0902 (6)	0.0498 (17)	
H13	0.6849	0.8275	0.0987	0.060*	
C14	0.7176 (9)	0.9729 (8)	0.0066 (6)	0.060 (2)	
H14	0.6715	0.9689	−0.0378	0.072*	
C15	0.7792 (9)	1.0608 (7)	−0.0092 (6)	0.060 (2)	
H15	0.7780	1.1167	−0.0656	0.072*	
C16	0.8444 (8)	1.0663 (5)	0.0601 (6)	0.0474 (17)	
C17	0.9158 (10)	1.1552 (7)	0.0493 (8)	0.062 (2)	
H17	0.9184	1.2130	−0.0062	0.075*	
C18	0.9789 (9)	1.1563 (6)	0.1182 (8)	0.063 (2)	
H18	1.0231	1.2148	0.1098	0.076*	
C19	0.9781 (7)	1.0697 (6)	0.2024 (7)	0.0468 (17)	
C20	1.0409 (9)	1.0668 (7)	0.2771 (8)	0.059 (2)	
H20	1.0872	1.1230	0.2717	0.071*	
C21	1.0327 (8)	0.9808 (8)	0.3570 (7)	0.056 (2)	
H21	1.0757	0.9774	0.4056	0.067*	
C22	0.9605 (7)	0.8977 (6)	0.3669 (6)	0.0443 (15)	
H22	0.9549	0.8405	0.4230	0.053*	
C23	0.9084 (6)	0.9817 (5)	0.2161 (5)	0.0373 (13)	
C24	0.8440 (6)	0.9794 (5)	0.1435 (5)	0.0374 (13)	
C25	0.5018 (7)	0.9451 (6)	0.3215 (6)	0.0445 (15)	
H25	0.5289	0.9676	0.2515	0.053*	
C26	0.3778 (8)	0.9917 (7)	0.3749 (7)	0.0548 (19)	
H26	0.3242	1.0447	0.3406	0.066*	
C27	0.3352 (7)	0.9605 (6)	0.4761 (7)	0.0524 (19)	
H27	0.2509	0.9902	0.5117	0.063*	
C28	0.4194 (7)	0.8820 (6)	0.5285 (6)	0.0434 (15)	
C29	0.3850 (8)	0.8462 (7)	0.6356 (6)	0.0514 (18)	
H29	0.3023	0.8739	0.6748	0.062*	
C30	0.4710 (8)	0.7722 (7)	0.6816 (6)	0.0528 (18)	
H30	0.4472	0.7517	0.7517	0.063*	
C31	0.5963 (7)	0.7258 (6)	0.6245 (5)	0.0436 (15)	
C32	0.6871 (8)	0.6473 (7)	0.6673 (6)	0.0530 (18)	
H32	0.6695	0.6252	0.7371	0.064*	
C33	0.8045 (9)	0.6025 (7)	0.6043 (6)	0.0548 (19)	
H33	0.8642	0.5482	0.6313	0.066*	
C34	0.8303 (7)	0.6406 (6)	0.5006 (5)	0.0451 (15)	
H34	0.9105	0.6125	0.4590	0.054*	
C35	0.6306 (6)	0.7583 (5)	0.5182 (5)	0.0363 (12)	
C36	0.5425 (6)	0.8397 (5)	0.4702 (5)	0.0359 (12)	

### Atomic displacement parameters (Å^2^)

**Table:**

	*U*^11^	*U*^22^	*U*^33^	*U*^12^	*U*^13^	*U*^23^
Re2	0.0577 (2)	0.05247 (19)	0.04822 (18)	−0.01579 (14)	−0.01504 (15)	−0.00749 (14)
Re1	0.0644 (2)	0.0581 (2)	0.03748 (16)	−0.00922 (15)	−0.00999 (14)	−0.01234 (13)
Co1	0.0324 (4)	0.0366 (4)	0.0285 (4)	−0.0069 (3)	−0.0071 (3)	−0.0067 (3)
N1	0.035 (2)	0.042 (3)	0.032 (2)	−0.007 (2)	−0.011 (2)	−0.008 (2)
N2	0.035 (2)	0.039 (3)	0.036 (3)	−0.012 (2)	−0.006 (2)	−0.005 (2)
N3	0.036 (2)	0.044 (3)	0.033 (3)	−0.007 (2)	−0.009 (2)	−0.005 (2)
N4	0.035 (2)	0.042 (3)	0.035 (3)	−0.007 (2)	−0.012 (2)	−0.012 (2)
N5	0.031 (2)	0.042 (3)	0.042 (3)	−0.003 (2)	−0.012 (2)	−0.010 (2)
N6	0.033 (2)	0.039 (3)	0.031 (2)	−0.007 (2)	−0.006 (2)	−0.006 (2)
O1	0.087 (5)	0.113 (6)	0.060 (4)	0.007 (5)	−0.019 (4)	−0.033 (4)
O2	0.165 (10)	0.136 (8)	0.082 (6)	−0.086 (7)	−0.030 (7)	−0.025 (6)
O3	0.219 (13)	0.079 (6)	0.077 (6)	−0.036 (7)	−0.055 (8)	0.007 (5)
O4	0.071 (5)	0.132 (9)	0.166 (11)	0.012 (6)	−0.023 (6)	−0.064 (8)
O5	0.087 (5)	0.063 (4)	0.113 (7)	−0.024 (4)	−0.033 (5)	0.001 (4)
O6	0.088 (6)	0.115 (7)	0.126 (8)	−0.038 (5)	−0.046 (6)	−0.027 (6)
O7	0.114 (8)	0.201 (12)	0.082 (7)	−0.069 (8)	−0.021 (6)	−0.011 (8)
O8	0.122 (7)	0.151 (9)	0.046 (4)	0.025 (7)	−0.025 (5)	−0.017 (5)
O9	0.141 (10)	0.137 (9)	0.087 (7)	−0.011 (8)	−0.042 (7)	−0.035 (7)
C1	0.036 (3)	0.055 (4)	0.044 (4)	−0.008 (3)	−0.014 (3)	−0.005 (3)
C2	0.037 (3)	0.054 (4)	0.046 (4)	−0.002 (3)	−0.006 (3)	−0.007 (3)
C3	0.042 (4)	0.051 (4)	0.055 (5)	0.006 (3)	−0.004 (3)	−0.010 (4)
C4	0.044 (3)	0.046 (4)	0.048 (4)	−0.005 (3)	−0.010 (3)	−0.014 (3)
C5	0.060 (5)	0.049 (4)	0.074 (6)	0.004 (4)	−0.009 (4)	−0.031 (4)
C6	0.070 (6)	0.066 (6)	0.091 (8)	−0.003 (5)	−0.017 (5)	−0.052 (6)
C7	0.054 (4)	0.046 (4)	0.048 (4)	−0.019 (3)	−0.011 (3)	−0.010 (3)
C8	0.063 (5)	0.059 (5)	0.071 (6)	−0.023 (4)	−0.026 (5)	−0.014 (4)
C9	0.047 (4)	0.059 (5)	0.079 (6)	−0.021 (4)	−0.009 (4)	−0.022 (5)
C10	0.039 (3)	0.055 (4)	0.049 (4)	−0.010 (3)	−0.008 (3)	−0.012 (3)
C11	0.036 (3)	0.034 (3)	0.038 (3)	−0.008 (2)	−0.010 (2)	−0.004 (2)
C12	0.036 (3)	0.032 (3)	0.031 (3)	−0.002 (2)	−0.008 (2)	−0.003 (2)
C13	0.055 (4)	0.060 (4)	0.036 (3)	−0.003 (3)	−0.016 (3)	−0.013 (3)
C14	0.061 (5)	0.079 (6)	0.039 (4)	0.003 (4)	−0.026 (4)	−0.008 (4)
C15	0.067 (5)	0.060 (5)	0.034 (4)	0.002 (4)	−0.012 (4)	0.009 (3)
C16	0.049 (4)	0.034 (3)	0.045 (4)	0.000 (3)	−0.006 (3)	0.001 (3)
C17	0.067 (5)	0.038 (4)	0.069 (6)	−0.010 (3)	−0.013 (5)	0.002 (4)
C18	0.054 (4)	0.040 (4)	0.088 (7)	−0.014 (3)	−0.007 (5)	−0.013 (4)
C19	0.036 (3)	0.041 (3)	0.061 (5)	−0.009 (3)	−0.003 (3)	−0.018 (3)
C20	0.049 (4)	0.057 (5)	0.077 (6)	−0.013 (4)	−0.012 (4)	−0.027 (5)
C21	0.041 (4)	0.074 (5)	0.067 (5)	−0.008 (3)	−0.019 (4)	−0.036 (5)
C22	0.040 (3)	0.054 (4)	0.045 (4)	−0.011 (3)	−0.013 (3)	−0.017 (3)
C23	0.027 (2)	0.036 (3)	0.044 (3)	−0.005 (2)	−0.003 (2)	−0.010 (3)
C24	0.034 (3)	0.040 (3)	0.033 (3)	−0.003 (2)	−0.006 (2)	−0.005 (2)
C25	0.044 (3)	0.043 (3)	0.047 (4)	−0.005 (3)	−0.019 (3)	−0.007 (3)
C26	0.039 (3)	0.056 (4)	0.068 (5)	−0.002 (3)	−0.018 (4)	−0.012 (4)
C27	0.034 (3)	0.054 (4)	0.069 (5)	0.004 (3)	−0.008 (3)	−0.028 (4)
C28	0.037 (3)	0.049 (4)	0.045 (4)	−0.010 (3)	−0.002 (3)	−0.019 (3)
C29	0.045 (4)	0.066 (5)	0.045 (4)	−0.016 (3)	0.002 (3)	−0.026 (4)
C30	0.051 (4)	0.070 (5)	0.035 (3)	−0.015 (4)	0.000 (3)	−0.019 (3)
C31	0.043 (3)	0.061 (4)	0.028 (3)	−0.018 (3)	−0.003 (3)	−0.012 (3)
C32	0.052 (4)	0.070 (5)	0.030 (3)	−0.022 (4)	−0.006 (3)	0.001 (3)
C33	0.053 (4)	0.056 (4)	0.047 (4)	−0.011 (3)	−0.020 (4)	0.011 (3)
C34	0.044 (3)	0.052 (4)	0.034 (3)	−0.005 (3)	−0.010 (3)	−0.003 (3)
C35	0.031 (3)	0.044 (3)	0.032 (3)	−0.012 (2)	−0.005 (2)	−0.004 (2)
C36	0.031 (3)	0.042 (3)	0.038 (3)	−0.008 (2)	−0.009 (2)	−0.012 (3)

### Geometric parameters (Å, °)

**Table:**

Re2—O7	1.549 (15)	C9—C10	1.407 (11)
Re2—O5	1.685 (8)	C9—H9	0.9300
Re2—O6	1.708 (8)	C10—H10	0.9300
Re2—O8	1.728 (8)	C11—C12	1.445 (9)
Re1—O3	1.688 (9)	C13—C14	1.395 (11)
Re1—O2	1.691 (9)	C13—H13	0.9300
Re1—O1	1.700 (7)	C14—C15	1.357 (14)
Re1—O4	1.724 (10)	C14—H14	0.9300
Co1—N6	2.122 (5)	C15—C16	1.397 (13)
Co1—N2	2.122 (5)	C15—H15	0.9300
Co1—N1	2.136 (5)	C16—C24	1.403 (9)
Co1—N4	2.147 (5)	C16—C17	1.450 (12)
Co1—N3	2.148 (6)	C17—C18	1.357 (15)
Co1—N5	2.151 (6)	C17—H17	0.9300
N1—C1	1.336 (8)	C18—C19	1.407 (12)
N1—C12	1.356 (8)	C18—H18	0.9300
N2—C10	1.318 (8)	C19—C20	1.414 (13)
N2—C11	1.362 (8)	C19—C23	1.417 (9)
N3—C13	1.317 (9)	C20—C21	1.359 (14)
N3—C24	1.352 (9)	C20—H20	0.9300
N4—C22	1.337 (9)	C21—C22	1.392 (11)
N4—C23	1.362 (9)	C21—H21	0.9300
N5—C25	1.336 (9)	C22—H22	0.9300
N5—C36	1.373 (9)	C23—C24	1.418 (10)
N6—C34	1.324 (9)	C25—C26	1.389 (11)
N6—C35	1.360 (8)	C25—H25	0.9300
O9—H109	0.85 (10)	C26—C27	1.342 (13)
O9—H209	0.86 (14)	C26—H26	0.9300
C1—C2	1.422 (11)	C27—C28	1.420 (11)
C1—H1	0.9300	C27—H27	0.9300
C2—C3	1.341 (12)	C28—C36	1.388 (9)
C2—H2	0.9300	C28—C29	1.424 (11)
C3—C4	1.407 (11)	C29—C30	1.358 (13)
C3—H3	0.9300	C29—H29	0.9300
C4—C12	1.411 (9)	C30—C31	1.418 (10)
C4—C5	1.417 (12)	C30—H30	0.9300
C5—C6	1.378 (13)	C31—C32	1.398 (11)
C5—H5	0.9300	C31—C35	1.410 (9)
C6—C7	1.418 (12)	C32—C33	1.397 (12)
C6—H6	0.9300	C32—H32	0.9300
C7—C8	1.390 (12)	C33—C34	1.386 (10)
C7—C11	1.396 (9)	C33—H33	0.9300
C8—C9	1.319 (13)	C34—H34	0.9300
C8—H8	0.9300	C35—C36	1.432 (9)
			
O7—Re2—O5	108.3 (5)	N2—C11—C12	116.7 (6)
O7—Re2—O6	113.2 (5)	C7—C11—C12	121.2 (6)
O5—Re2—O6	109.3 (5)	N1—C12—C4	123.8 (6)
O7—Re2—O8	109.7 (5)	N1—C12—C11	117.5 (5)
O5—Re2—O8	107.7 (6)	C4—C12—C11	118.7 (6)
O6—Re2—O8	108.5 (5)	N3—C13—C14	122.7 (8)
O3—Re1—O2	111.0 (5)	N3—C13—H13	118.7
O3—Re1—O1	107.9 (5)	C14—C13—H13	118.7
O2—Re1—O1	111.5 (5)	C15—C14—C13	119.4 (8)
O3—Re1—O4	108.4 (6)	C15—C14—H14	120.3
O2—Re1—O4	110.6 (6)	C13—C14—H14	120.3
O1—Re1—O4	107.4 (5)	C14—C15—C16	119.3 (7)
N6—Co1—N2	94.4 (2)	C14—C15—H15	120.3
N6—Co1—N1	101.4 (2)	C16—C15—H15	120.3
N2—Co1—N1	78.1 (2)	C15—C16—C24	118.1 (7)
N6—Co1—N4	93.0 (2)	C15—C16—C17	123.8 (8)
N2—Co1—N4	169.5 (2)	C24—C16—C17	118.1 (8)
N1—Co1—N4	93.2 (2)	C18—C17—C16	121.6 (8)
N6—Co1—N3	163.7 (2)	C18—C17—H17	119.2
N2—Co1—N3	96.8 (2)	C16—C17—H17	119.2
N1—Co1—N3	92.5 (2)	C17—C18—C19	120.6 (8)
N4—Co1—N3	77.5 (2)	C17—C18—H18	119.7
N6—Co1—N5	78.0 (2)	C19—C18—H18	119.7
N2—Co1—N5	94.3 (2)	C18—C19—C20	123.1 (7)
N1—Co1—N5	172.3 (2)	C18—C19—C23	119.6 (8)
N4—Co1—N5	94.5 (2)	C20—C19—C23	117.3 (8)
N3—Co1—N5	89.4 (2)	C21—C20—C19	119.1 (7)
C1—N1—C12	117.7 (6)	C21—C20—H20	120.5
C1—N1—Co1	129.0 (5)	C19—C20—H20	120.5
C12—N1—Co1	113.1 (4)	C20—C21—C22	120.7 (8)
C10—N2—C11	117.6 (6)	C20—C21—H21	119.7
C10—N2—Co1	128.5 (5)	C22—C21—H21	119.7
C11—N2—Co1	113.8 (4)	N4—C22—C21	122.1 (8)
C13—N3—C24	118.8 (6)	N4—C22—H22	119.0
C13—N3—Co1	127.8 (5)	C21—C22—H22	119.0
C24—N3—Co1	113.0 (4)	N4—C23—C19	122.3 (7)
C22—N4—C23	118.5 (6)	N4—C23—C24	117.6 (6)
C22—N4—Co1	128.4 (5)	C19—C23—C24	120.1 (7)
C23—N4—Co1	113.1 (4)	N3—C24—C16	121.7 (7)
C25—N5—C36	117.2 (6)	N3—C24—C23	118.2 (6)
C25—N5—Co1	129.7 (5)	C16—C24—C23	120.1 (7)
C36—N5—Co1	113.1 (4)	N5—C25—C26	122.8 (8)
C34—N6—C35	118.8 (6)	N5—C25—H25	118.6
C34—N6—Co1	127.0 (5)	C26—C25—H25	118.6
C35—N6—Co1	114.2 (4)	C27—C26—C25	120.1 (8)
H109—O9—H209	116 (16)	C27—C26—H26	119.9
N1—C1—C2	121.7 (7)	C25—C26—H26	119.9
N1—C1—H1	119.1	C26—C27—C28	119.7 (7)
C2—C1—H1	119.1	C26—C27—H27	120.1
C3—C2—C1	120.1 (7)	C28—C27—H27	120.1
C3—C2—H2	120.0	C36—C28—C27	117.0 (7)
C1—C2—H2	120.0	C36—C28—C29	119.2 (7)
C2—C3—C4	120.2 (7)	C27—C28—C29	123.9 (7)
C2—C3—H3	119.9	C30—C29—C28	121.2 (7)
C4—C3—H3	119.9	C30—C29—H29	119.4
C3—C4—C12	116.5 (7)	C28—C29—H29	119.4
C3—C4—C5	123.6 (7)	C29—C30—C31	121.1 (7)
C12—C4—C5	120.0 (7)	C29—C30—H30	119.4
C6—C5—C4	119.8 (7)	C31—C30—H30	119.4
C6—C5—H5	120.1	C32—C31—C35	117.7 (7)
C4—C5—H5	120.1	C32—C31—C30	123.8 (7)
C5—C6—C7	122.6 (8)	C35—C31—C30	118.5 (7)
C5—C6—H6	118.7	C33—C32—C31	119.4 (7)
C7—C6—H6	118.7	C33—C32—H32	120.3
C8—C7—C11	117.4 (8)	C31—C32—H32	120.3
C8—C7—C6	124.7 (8)	C34—C33—C32	118.7 (7)
C11—C7—C6	117.8 (7)	C34—C33—H33	120.6
C9—C8—C7	120.7 (8)	C32—C33—H33	120.6
C9—C8—H8	119.6	N6—C34—C33	123.2 (7)
C7—C8—H8	119.6	N6—C34—H34	118.4
C8—C9—C10	119.2 (8)	C33—C34—H34	118.4
C8—C9—H9	120.4	N6—C35—C31	122.2 (6)
C10—C9—H9	120.4	N6—C35—C36	117.7 (6)
N2—C10—C9	122.7 (8)	C31—C35—C36	120.1 (6)
N2—C10—H10	118.7	N5—C36—C28	123.2 (6)
C9—C10—H10	118.7	N5—C36—C35	117.0 (6)
N2—C11—C7	122.1 (6)	C28—C36—C35	119.8 (6)
			
N6—Co1—N1—C1	−86.0 (6)	Co1—N1—C12—C4	175.3 (6)
N2—Co1—N1—C1	−178.2 (6)	C1—N1—C12—C11	177.9 (6)
N4—Co1—N1—C1	7.8 (6)	Co1—N1—C12—C11	−7.4 (7)
N3—Co1—N1—C1	85.4 (6)	C3—C4—C12—N1	−2.7 (11)
N5—Co1—N1—C1	−170.6 (15)	C5—C4—C12—N1	177.6 (7)
N6—Co1—N1—C12	100.1 (4)	C3—C4—C12—C11	−179.9 (7)
N2—Co1—N1—C12	7.9 (4)	C5—C4—C12—C11	0.4 (11)
N4—Co1—N1—C12	−166.2 (4)	N2—C11—C12—N1	1.2 (9)
N3—Co1—N1—C12	−88.6 (4)	C7—C11—C12—N1	−178.3 (6)
N5—Co1—N1—C12	15.5 (19)	N2—C11—C12—C4	178.6 (6)
N6—Co1—N2—C10	75.1 (7)	C7—C11—C12—C4	−0.9 (10)
N1—Co1—N2—C10	175.8 (7)	C24—N3—C13—C14	−2.1 (11)
N4—Co1—N2—C10	−149.6 (11)	Co1—N3—C13—C14	169.5 (6)
N3—Co1—N2—C10	−93.0 (7)	N3—C13—C14—C15	2.8 (13)
N5—Co1—N2—C10	−3.2 (7)	C13—C14—C15—C16	−1.8 (13)
N6—Co1—N2—C11	−108.0 (5)	C14—C15—C16—C24	0.4 (12)
N1—Co1—N2—C11	−7.3 (5)	C14—C15—C16—C17	178.6 (8)
N4—Co1—N2—C11	27.3 (14)	C15—C16—C17—C18	−179.3 (9)
N3—Co1—N2—C11	83.8 (5)	C24—C16—C17—C18	−1.1 (13)
N5—Co1—N2—C11	173.7 (5)	C16—C17—C18—C19	0.7 (14)
N6—Co1—N3—C13	−123.2 (8)	C17—C18—C19—C20	−179.4 (9)
N2—Co1—N3—C13	10.0 (6)	C17—C18—C19—C23	−1.2 (12)
N1—Co1—N3—C13	88.3 (6)	C18—C19—C20—C21	178.7 (8)
N4—Co1—N3—C13	−179.0 (6)	C23—C19—C20—C21	0.5 (11)
N5—Co1—N3—C13	−84.2 (6)	C19—C20—C21—C22	−1.6 (12)
N6—Co1—N3—C24	48.9 (9)	C23—N4—C22—C21	0.3 (10)
N2—Co1—N3—C24	−177.9 (4)	Co1—N4—C22—C21	−177.0 (5)
N1—Co1—N3—C24	−99.6 (5)	C20—C21—C22—N4	1.3 (12)
N4—Co1—N3—C24	−6.9 (4)	C22—N4—C23—C19	−1.5 (9)
N5—Co1—N3—C24	87.8 (5)	Co1—N4—C23—C19	176.2 (5)
N6—Co1—N4—C22	16.2 (6)	C22—N4—C23—C24	179.2 (6)
N2—Co1—N4—C22	−119.2 (12)	Co1—N4—C23—C24	−3.1 (7)
N1—Co1—N4—C22	−85.4 (6)	C18—C19—C23—N4	−177.2 (7)
N3—Co1—N4—C22	−177.2 (6)	C20—C19—C23—N4	1.1 (10)
N5—Co1—N4—C22	94.4 (6)	C18—C19—C23—C24	2.2 (10)
N6—Co1—N4—C23	−161.2 (4)	C20—C19—C23—C24	−179.6 (6)
N2—Co1—N4—C23	63.4 (13)	C13—N3—C24—C16	0.6 (10)
N1—Co1—N4—C23	97.2 (4)	Co1—N3—C24—C16	−172.2 (5)
N3—Co1—N4—C23	5.3 (4)	C13—N3—C24—C23	−179.5 (6)
N5—Co1—N4—C23	−83.0 (4)	Co1—N3—C24—C23	7.7 (7)
N6—Co1—N5—C25	−179.1 (6)	C15—C16—C24—N3	0.2 (11)
N2—Co1—N5—C25	−85.5 (6)	C17—C16—C24—N3	−178.1 (7)
N1—Co1—N5—C25	−93.0 (18)	C15—C16—C24—C23	−179.7 (7)
N4—Co1—N5—C25	88.7 (6)	C17—C16—C24—C23	2.0 (10)
N3—Co1—N5—C25	11.3 (6)	N4—C23—C24—N3	−3.1 (9)
N6—Co1—N5—C36	0.7 (4)	C19—C23—C24—N3	177.5 (6)
N2—Co1—N5—C36	94.3 (5)	N4—C23—C24—C16	176.8 (6)
N1—Co1—N5—C36	86.8 (17)	C19—C23—C24—C16	−2.6 (9)
N4—Co1—N5—C36	−91.5 (5)	C36—N5—C25—C26	−1.6 (11)
N3—Co1—N5—C36	−168.9 (5)	Co1—N5—C25—C26	178.1 (6)
N2—Co1—N6—C34	88.4 (6)	N5—C25—C26—C27	−0.4 (13)
N1—Co1—N6—C34	9.6 (6)	C25—C26—C27—C28	1.9 (13)
N4—Co1—N6—C34	−84.2 (6)	C26—C27—C28—C36	−1.2 (11)
N3—Co1—N6—C34	−138.2 (8)	C26—C27—C28—C29	178.0 (8)
N5—Co1—N6—C34	−178.2 (6)	C36—C28—C29—C30	0.5 (11)
N2—Co1—N6—C35	−94.8 (5)	C27—C28—C29—C30	−178.7 (8)
N1—Co1—N6—C35	−173.6 (4)	C28—C29—C30—C31	−1.8 (12)
N4—Co1—N6—C35	92.6 (5)	C29—C30—C31—C32	−178.2 (8)
N3—Co1—N6—C35	38.6 (10)	C29—C30—C31—C35	0.2 (12)
N5—Co1—N6—C35	−1.4 (4)	C35—C31—C32—C33	−1.8 (11)
C12—N1—C1—C2	1.9 (10)	C30—C31—C32—C33	176.6 (8)
Co1—N1—C1—C2	−171.8 (5)	C31—C32—C33—C34	2.6 (12)
N1—C1—C2—C3	−2.3 (12)	C35—N6—C34—C33	1.7 (11)
C1—C2—C3—C4	0.0 (13)	Co1—N6—C34—C33	178.4 (6)
C2—C3—C4—C12	2.3 (12)	C32—C33—C34—N6	−2.6 (13)
C2—C3—C4—C5	−178.0 (9)	C34—N6—C35—C31	−0.8 (10)
C3—C4—C5—C6	−179.8 (10)	Co1—N6—C35—C31	−177.9 (5)
C12—C4—C5—C6	−0.2 (14)	C34—N6—C35—C36	179.0 (6)
C4—C5—C6—C7	0.4 (17)	Co1—N6—C35—C36	1.9 (7)
C5—C6—C7—C8	−178.6 (10)	C32—C31—C35—N6	0.9 (10)
C5—C6—C7—C11	−1.0 (16)	C30—C31—C35—N6	−177.6 (7)
C11—C7—C8—C9	−3.0 (14)	C32—C31—C35—C36	−178.9 (7)
C6—C7—C8—C9	174.6 (10)	C30—C31—C35—C36	2.6 (10)
C7—C8—C9—C10	4.2 (16)	C25—N5—C36—C28	2.3 (10)
C11—N2—C10—C9	−1.3 (12)	Co1—N5—C36—C28	−177.5 (5)
Co1—N2—C10—C9	175.5 (7)	C25—N5—C36—C35	179.9 (6)
C8—C9—C10—N2	−2.1 (15)	Co1—N5—C36—C35	0.1 (7)
C10—N2—C11—C7	2.5 (10)	C27—C28—C36—N5	−0.9 (10)
Co1—N2—C11—C7	−174.7 (5)	C29—C28—C36—N5	179.8 (7)
C10—N2—C11—C12	−177.0 (6)	C27—C28—C36—C35	−178.5 (6)
Co1—N2—C11—C12	5.8 (7)	C29—C28—C36—C35	2.2 (10)
C8—C7—C11—N2	−0.5 (11)	N6—C35—C36—N5	−1.4 (9)
C6—C7—C11—N2	−178.3 (8)	C31—C35—C36—N5	178.5 (6)
C8—C7—C11—C12	179.0 (7)	N6—C35—C36—C28	176.3 (6)
C6—C7—C11—C12	1.2 (12)	C31—C35—C36—C28	−3.8 (10)
C1—N1—C12—C4	0.6 (10)		

### Hydrogen-bond geometry (Å, °)

**Table:**

*D*—H···*A*	*D*—H	H···*A*	*D*···*A*	*D*—H···*A*
O9—H209···O1^i^	0.85 (10)	1.99 (8)	2.802 (13)	157 (19)
O9—H109···O8	0.86 (14)	2.26 (17)	2.953 (17)	139 (23)
C15—H15···O9^ii^	0.93	2.56	3.271 (14)	134
C33—H33···O5^iii^	0.93	2.51	3.116 (12)	123
C5—H5···O4^iv^	0.93	2.45	3.309 (13)	154
C33—H33···O5^iii^	0.93	2.51	3.116 (12)	123

Symmetry codes: (i) *x*, *y*, *z*+1; (ii) *x*, *y*+1, *z*−1; (iii) −*x*+2, −*y*+1, −*z*+1; (iv) *x*+1, *y*, *z*.
